# Supplementary figures and images for: An experimental erythrocyte rigidity index (Ri) and its correlations with Transcranial Doppler velocities (TAMMV), Gosling Pulsatility Index PI, hematocrit, hemoglobin concentration and red cell distribution width (RDW)
Source: PLoS One. 2020 Feb 21;15(2):e0229105. doi: 10.1371/journal.pone.0229105 (PMC7034921; doi:10.1371/journal.pone.0229105)

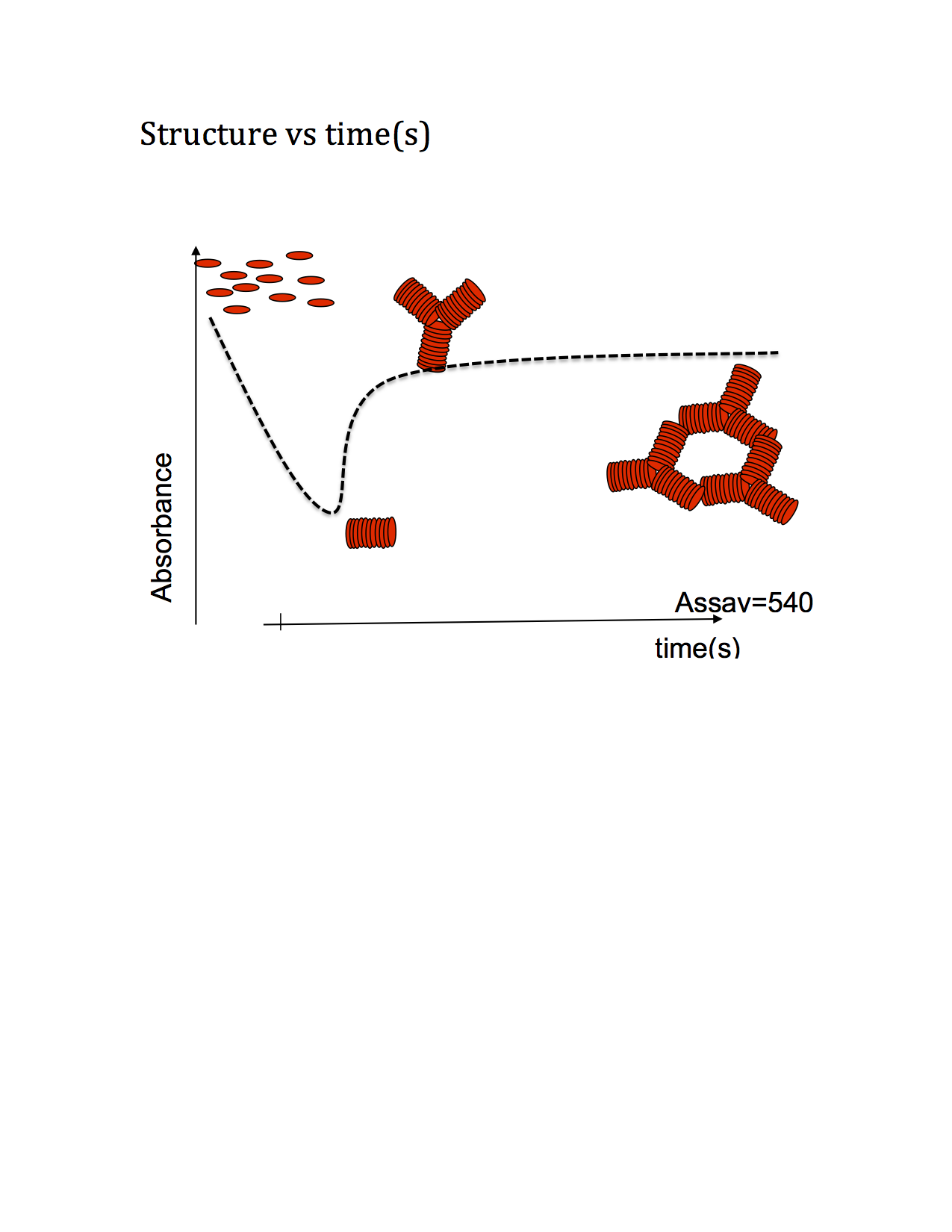

Supplement: S1 Fig — (TIFF) [file pone.0229105.s005.tiff]

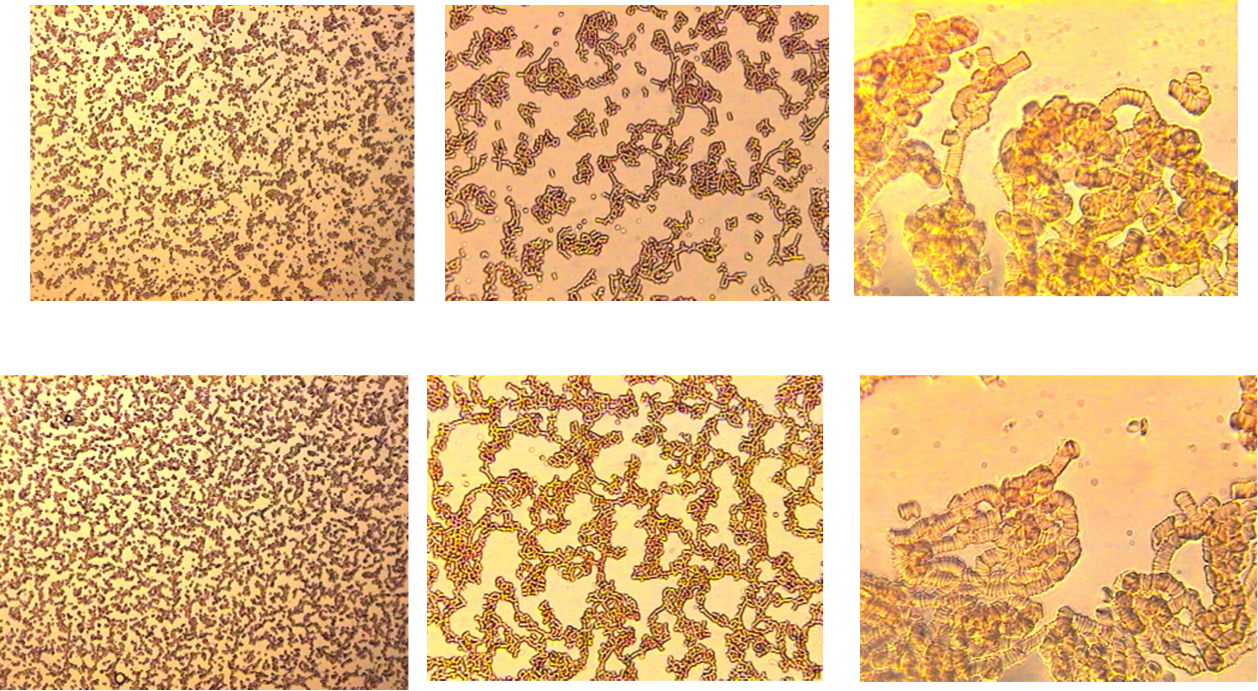

Supplement: S2 Fig — DONOR: No HU (top row) and HU-added (bottom row) microstructure of blood samples obtained by light microscopy. (TIF) [file pone.0229105.s006.tif]

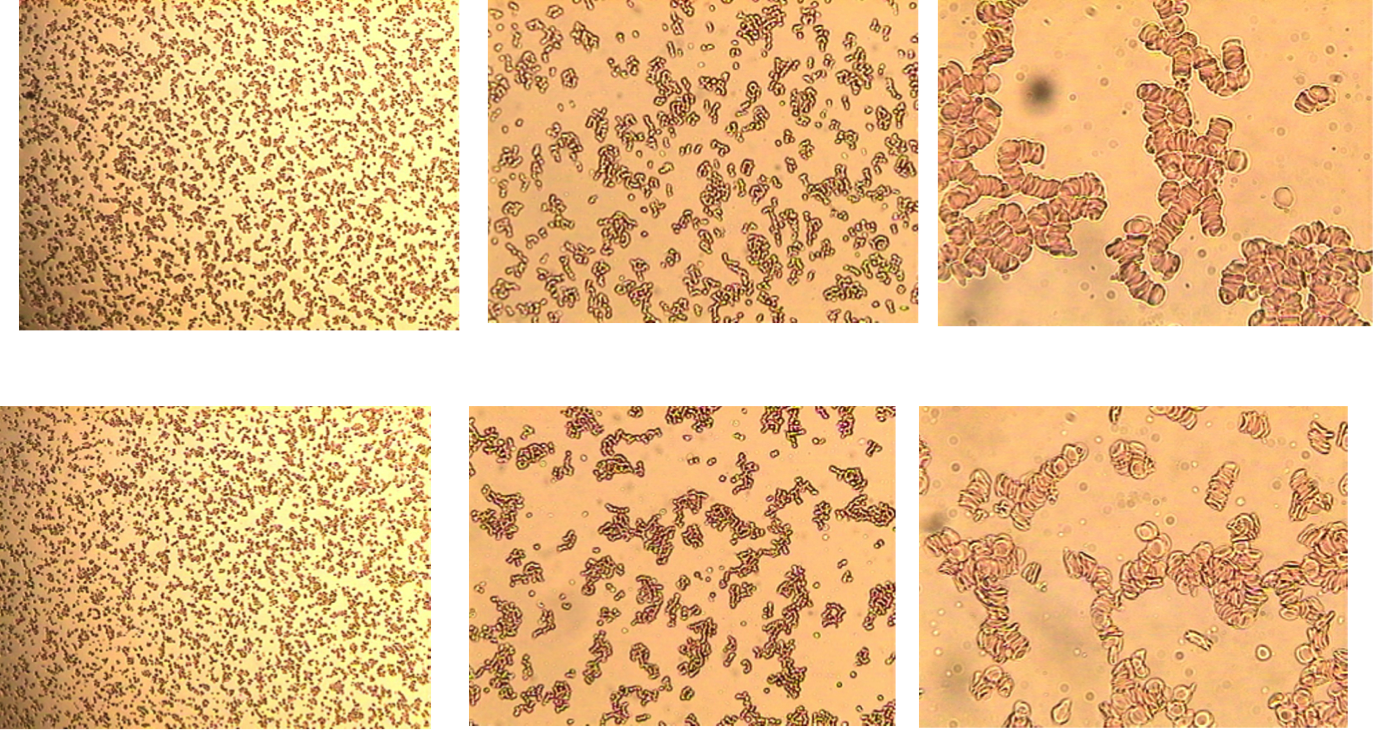

Supplement: S3 Fig — PATIENT: No HU (top row) and HU-added (bottom row) microstructure of blood samples obtained by light microscopy. (TIF) [file pone.0229105.s007.tif]

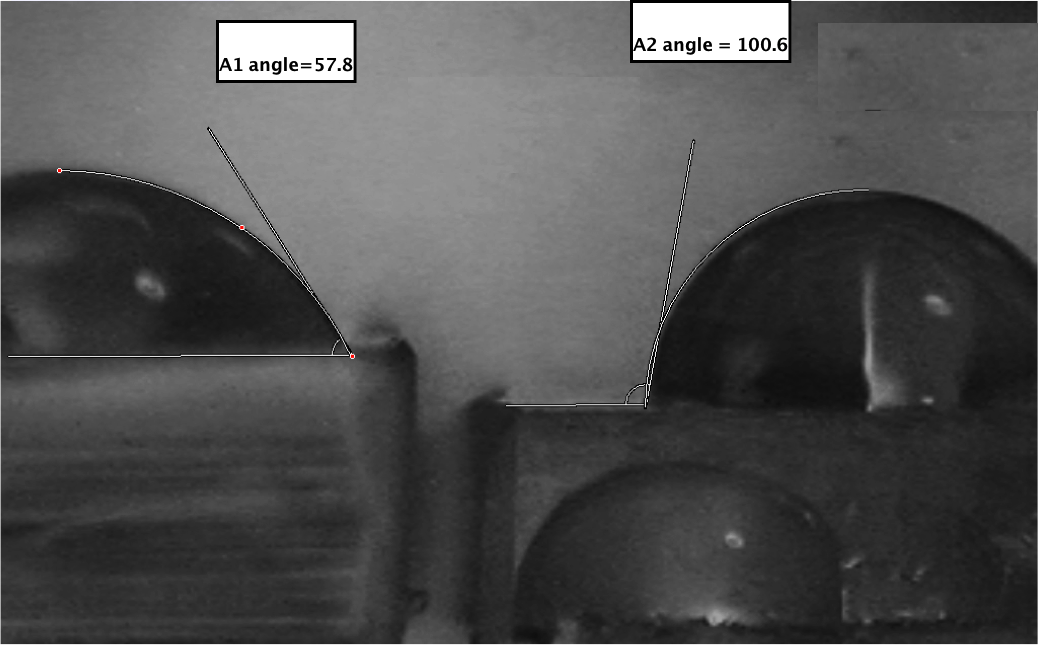

Supplement: S4 Fig — (TIF) [file pone.0229105.s008.tif]
